# Dimethyl Sulfoxide Dentin Pretreatments Do Not Improve Bonding of a Universal Adhesive in Etch-and-Rinse or Self-etch Modes

**DOI:** 10.3290/j.jad.b2701705

**Published:** 2022-03-01

**Authors:** Rodrigo Mafra Magalhães de Mello, Bárbara Albertini Roquim Alcântara, Fabiana Mantovani Gomes França, Flávia Lucisano Botelho do Amaral, Roberta Tarkany Basting

**Affiliations:** a MSc Student, Faculdade São Leopoldo Mandic, Campinas, Brazil. Data acquisition, interpreted the data and wrote the paper.; b MSc Student, Faculdade São Leopoldo Mandic, Campinas, Brazil. Performed data acquisition, interpreted the data, wrote the manuscript.; c Professor, Faculdade São Leopoldo Mandic, Campinas, Brazil. Revised the paper, wrote the manuscript, approved the final version.; d Professor, Faculdade São Leopoldo Mandic, Campinas, Brazil. Data analysis, revised the paper carefully for important intellectual content, approved the final version.; e Professor, Faculdade São Leopoldo Mandic, Campinas, Brazil. Study concept and design, interpretated the data, and wrote the paper.

**Keywords:** aging, ethanol, hybrid layer, micromorphology, microtensile bond strength

## Abstract

**Purpose:**

To evaluate the influence of dimethyl sulfoxide (DMSO) solutions used as dentin pretreatments on microtensile bond strength (µTBS), as well as the dentin/restoration interface micromorphology of a universal adhesive in etch-and-rinse or self-etch mode.

**Materials and Methods:**

Eighty blocks of dentin were submitted to acid conditioning with 35% phosphoric acid (etch-and-rinse), or not (self-etch), and distributed among the treatments (n = 10): CON: Scotchbond Universal/3M Oral Care; DMSO: pretreatment with DMSO; DMSO/water: pretreatment with DMSO in water (1:1); DMSO/ethanol: pretreatment with DMSO in ethanol (1:1). Microtensile bond strength and failure tests were performed after 24-h and 6-month storage. The tooth-restoration interface was evaluated using scanning electron microscopy to assess the hybrid layer formed.

**Results:**

The interaction between treatments, storage time, and etching modes was not significant for µTBS (p = 0.2469). The DMSO, DMSO/water and DMSO/ethanol pretreatments did not affect µTBS values at either time point (p = 0.8732). Aging decreased µTBS over time only for the etch-and-rinse strategy, although the groups presented higher microtensile bond strengths in etch-and-rinse mode than in self-etch mode at both time points (p < 0.0001). The micromorphological images of the interface showed that different DMSO pretreatment solutions did not impair hybrid layer formation.

**Conclusion:**

The use of dentin pretreatments containing DMSO did not improve the bonding or the micromorphology of a universal adhesive in etch-and-rinse or self-etch modes.

Universal adhesives are composed of acidic, hydrophobic, and solvent hydrophilic monomers that enable their use in multiple modes (etch-and-rinse, self-etch, or selective enamel-etching strategies)^14,32^ and make them very versatile.^14^ The solvents composing universal adhesives can be water, ethanol, acetone or combinations of these, all of which allow acid monomer ionization and interaction of the solvents with dental substrates.^8,23,45^ These two factors lead to the displacement of water from the collagen fibril network, and the infiltration of resin monomers in the interfibrillar dentin.^1^ Ethanol, in particular, allows greater displacement of water due to its higher vapor pressure (40 mmHg) in relation to water (17 mmHg).^6^ Residual water is known to trigger the hydrolytic degradation of polymers and collagen, hastened by the acidic pH of the monomer,^12,31^ and leading to degradation of the hybrid layer.^11^

Although dimethyl sulfoxide solvent (DMSO) is not contained in adhesives, it can solubilize a wide variety of polar and nonpolar compounds,^20^ and also displace a higher volume of water than ethanol or water, owing to its vapor pressure of 0.6 mmHg.^43^ DMSO used as a dentin pretreatment in the wet-bonding technique is a non-toxic substance that enhances the ability of the adhesive monomer to penetrate the dentin matrix,^21,36^ improves the wettability of demineralized dentin,^11^ and inhibits dentin metalloproteinase activity.^34,39^ Dentin pretreatment with 50% DMSO in an aqueous solution has been reported to increase the encapsulation of collagen fibers when a conventional or a self-etching adhesive is used.^36^ This improves the quality of the collagen-resin interface for a period of up to 2 years, without impairing adhesive conversion or reducing nanoleakage.^37^ DMSO used either pure or solubilized at 50% in distilled water or ethanol produces a hybrid layer with higher integrity and fewer sites vulnerable to degradation.^13,34,35^ Even when DMSO was solubilized in water at a reduced concentration (0.004%), its use as a pretreatment led to higher bond strengths after 6 months of storage^39^ when using a conventional two-step adhesive. Salim Al-Ani et al^28^ also showed that the use of DMSO as pretreatment in concentrations between 0.001% and 20% in an aqueous solution promoted the formation of a hybrid layer with greater bond durability, and its incorporation (0.2% or 2%) into simplified etch-and-rinse adhesive maintained the long-term stability of the dentin bond.^38^

When using universal adhesives, the self-etch strategy benefits from 10-MDP phosphate monomer.^16,40^ This monomer promotes chemical adhesion of the system to the substrate due to the interaction between 10-MDP of the phosphate group and the calcium from hydroxyapatite; the product forms a stable salt.^8,23,45^ In this strategy, an increase in dentin permeability is avoided, although it is recognized that universal adhesives behave as semi-permeable membranes that allow dentin fluid to pass to the adhesive interface.^19,42^ In this regard, pretreatment with 50% DMSO in ethanol using a universal adhesive in the self-etching mode allowed formation of a hybrid layer less vulnerable to degradation.^35^

However, the etch-and-rinse strategy of a universal adhesive can be used on dentin with removal of the smear layer to allow greater penetration of the adhesive in the dentinal tubules. This process produces thicker hybrid layers, longer and more numerous resin tags,^46^ and even greater bond strength, compared with the self-etch strategy.^10^ Therefore, DMSO has the potential to foster greater penetration of resin monomers into a previously demineralized dentin surface, whether solubilized in water or ethanol, or not solubilized. In this regard, the present study aims to investigate the influence of dentin pretreatments with DMSO on dentin bond strength, according to different solubilization media, and two different bonding strategies (etch-and-rinse and self-etch) of a universal adhesive. The null hypotheses to be studied are that pretreatments with DMSO (whether solubilized in water or ethanol, or not solubilized), associated with different bonding strategies of a universal adhesive (etch-and-rinse or self-etch) to dentin would have no effect on: 1) bond strength to dentin; 2) longevity of bond strength; or 3) failure mode.

## Materials And Methods

### Specification of Materials, Producing DMSO Solutions

Table 1 shows the materials used in the present study, their composition, and methods of use. DMSO was solubilized in distilled water or ethanol by diluting it in these solutions to obtain a final concentration of 50% (v/v). The rationale for using 50% (v/v) DMSO was based on three studies by Stape et al^35–37^ which reported significant improvements in resin-dentin interactions when using conventional adhesives. The molecular weight was considered for calculating the appropriate concentration. DMSO and distilled water or ethanol were added to an Eppendorf tube with a pipette, vortexed for 1 min, and used promptly. The pH of the solutions was measured in triplicate with a microelectrode (Model 2A14, Analyser Instrumentação Analítica; São Paulo, SP, Brazil) and a pH meter (Model MPA 210, MS Tecnopon Instrumentação; Piracicaba, SP, Brazil), obtaining pH values of 10.52 for DMSO, 6.64 for DMSO solubilized in water, and 8.74 for DMSO solubilized in ethanol. The pH of the universal adhesive was measured to be pH 2.92.

**Table 1:** Materials used, composition and protocol of use

Material (manufacturer, city, state, country) / Batch number	Composition (wt%)	Application protocol
Scotchbond Universal (3M Oral Care; St Paul, MN, USA)/ 1926600462	2-hydroxyethyl methacrylate (15–25), bis-GMA (15–25), ethanol (10–15), water (10–15), 1,10-decanediol phosphate methacrylate (1–10), acrylic copolymer and itaconic acid (1–5), camphorquinone (< 2) and N, N-dimethylbenzocaine (< 2)*	Apply the adhesive for 20 s (active application); apply a gentle jet of air over the adhesive for 5 s (solvent evaporation); photoactivate for 10 s.
Phosphoric acid 35% – Ultra-Etch (Ultradent Products, St. Jordan, Utah, USA)/ D00ZI	Phosphoric acid (< 40), aluminum cobalt blue spinel (< 1) and siloxane (< 1) *	Apply for 15 s; rinse for 30 S; dry with absorbent paper.
DMSO 99.5% (Valhoma; Tulsa, Oklahoma, USA)/SHBK9394	Molecular formula: (CH_3_)_2_SO Molecular weight: 7.13 g/mol	Under light pressure, apply in circular scrubbing movements using the disposable applicator for 60 s (Stape et al^35–37^); remove excess with absorbent paper.
Ethanol 99.5% P.A. (Anidrol Produtos para Laboratórios; Diadema, SP)/32526	Molecular formula: (C_2_H_6_O) Molecular weight: 46.07 g/mol	--
Resina Filtek Z350 XT (3M Oral Care), A2 Body Shade/ 1927000401, 1812800687, 1819400353	Treated silanized ceramic (60–80), silane treated silica (1–10), UDMA (1–10), bisphenol A polyethylene glycol dimethacrylate diether (1–10), bis-GMA (1–10), zirconia ceramic (66402-68-4), surface modified with 3-methacryloxypropyltrimethoxysilane (2530-85-0), bulk material (1.96–5), polyethylene glycol dimethacrylate (< 5) and dimethacrylate triethylene glycol (0.2364)*	Increment thickness of 2 mm; photoactivation for 20 s each layer.

Bis-GMA: bisphenol A diglycidyl ether dimethacrylate; UDMA: dimethacrylate diurethane. *Material Safety Data Sheet of the manufacturer.

### Selection of Teeth, Dentin Blocks, Dentin Pretreatments

After approval of the study by the local ethics committee (CAAE 12345519.2.0000.5374/29386619.9.0000.5374), 80 sound, recently extracted human third molars without cracks or any changes in enamel and/or dentin were selected and frozen immediately afterwards. The specimens were obtained by cleaning the teeth externally with periodontal curettes, sectioning the teeth perpendicular to their long axis, and removing the enamel from the occlusal surface to obtain a flat surface of superficial dentin. Next, the surfaces were polished with 600-grit silicon carbide paper disks in a rotating electric polisher (Aropol 2V, Arotec; Cotia, SP, Brazil) to obtain a standardized surface with smear layer formation. The specimens were sectioned again 3 mm below the cementoenamel junction, exposing the pulp chamber. The pulp was cleaned with dentin excavators, and an adhesive (Scotchbond Universal, 3M Oral Care; St. Paul, MN, USA) was applied in the pulp chamber, light polymerizsed with an LED photocuring device (Valo Cordless, Ultradent; South Jordan, UT, USA) and filled with resin composite (Filtek Z350 XT, 3M Oral Care).

The dentin blocks were randomly distributed into eight groups to apply the treatments (n = 10). The etch-and-rinse adhesive strategy was used in four of these groups by applying phosphoric acid and the universal adhesive according to the manufacturer’s instructions (Table 1). In the other four groups, the self-etch adhesive strategy was used. The groups received the following treatments for each adhesive strategy:

- Control (CON): the universal adhesive was applied according to the manufacturer’s instructions (Table 1). Photoactivation was performed with an LED photocuring device (Valo Cordless, Ultradent), in standard mode with 1000 mW/cm^2^.
- Pretreatment with DMSO (DMSO): DMSO was applied with a disposable applicator (Micro Brush, KG Sorensen; Cotia, SP, Brazil) for 60 s, prior to the application of the universal adhesive.^35–37^ The excess was removed with absorbent paper, and the adhesive was applied.
- Pretreatment with DMSO solubilized in water (DMSO/water): DMSO was applied with a disposable applicator (Micro Brush) for 60 s, prior to the application of the universal adhesive.^35–37^ The excess was removed with absorbent paper, and the adhesive was applied.
- Pretreatment with DMSO solubilized in ethanol (DMSO/ethanol): DMSO was applied with a disposable applicator (Micro Brush) for 60 s, prior to the application of the universal adhesive.^35–37^ The excess was removed with absorbent paper, and the adhesive was applied.

Next, the resin composite was inserted using the incremental technique with increments of up to 2 mm, then light polymerized with the LED curing device (Valo Cordless), in standard mode with 1000 mW/cm^2^. Blocks of 5 mm x 5 mm were obtained using a nanohybrid composite (Filtek Z350 XT, shade A2B, 3M Oral Care).

### Microtensile Bond Strength Tests

The teeth were stored under humid conditions in an incubator at 37°C for 24 h, and then sectioned perpendicular to the dentin/resin interface with a double-sided diamond disk in a cutting machine (Isomet 1000, Buehler; Lake Bluff, IL, USA) to obtain stick-shaped specimns. A slice of each tooth was selected for micromorphological evaluation of the dentin/resin interface.

The resulting sticks were stored immersed in simulated body fluid (SBF) solution containing 50 mmol/l HEPES, 5 mmol/l CaCl_2_ 2H_2_O, 0.001 mmol/l ZnCl_2_, 150 mmol/l NaCl, and 3 mmol/l sodium azide at pH 7.4,^9^ in an incubator at 37°C, which was changed weekly. The bonding area of the sticks in mm^2^ was measured using a digital caliper (Mitutoyo Sul Americana; Suzano, SP, Brazil). Half of the sticks obtained from each tooth were evaluated after 24 h, while the other half were evaluated after 6 months of storage.

The sticks were fixed in a metallic device with cyanoacrylate glue (Super Bonder; Itapevi, São Paulo, Brazil), and subjected to microtensile bond strength testing in a universal testing machine (EZ Test, Shimadzu; Kyoto, Japan) with a 50-N load cell at a speed of 0.5 mm/min until fracture. The microtensile bond strength by group was obtained by averaging the values measured on different sticks obtained from the same tooth, according to the interface area, using the following formula: microtensile bond strength (in MPa) = load (in N)/area (in mm^2^).

### Fracture Mode Analysis

The surfaces fractured after the microtensile bond strength tests were evaluated using a stereoscopic magnifying glass (EK3ST, Eikonal Equip Óticos e Analíticos; São Paulo, SP, Brazil) at 40X magnification to determine the fracture mode. The fractures were classified as adhesive, cohesive in dentin, cohesive in resin, or mixed.

### Micromorphological Analysis of the Dentin/Resin Interface

The slices of each tooth obtained after cutting the resin/dentin interface were prepared for SEM micromorphological analysis. The surfaces were polished with sandpaper of decreasing abrasive granulations (600- and 1200-grit) (Imperial Wetordry, 3M Oral Care), then felt and polishing pastes of four different granulations. The surfaces were copiously rinsed, then demineralized for 30 s with 6N hydrochloric acid (HCl), and washed again. Then they were deproteinized with 3% sodium hypochlorite solution for 10 min, followed by washing with distilled water for 15 s, drying with absorbent paper, and dehydration in an ascending series of ethanol concentrations (25%, 50%, 75% and 100%).^27^

The slices were sputter-coated with gold for 60 s and examined in a scanning electron microscope (Leo 440i, LEO Electron Microscopy; Oxford, Cambridge, UK), at a voltage of 10 Kv and 2000X magnification. Differences in hybrid layer formation were evaluated descriptively according to the groups.

### Statistical Analysis

The data were analyzed using the R program.^26^ Initially, descriptive and exploratory analyses were performed. Since the microtensile bond strength data did not meet the assumptions for parametric analysis, generalized linear models were used, considering the design of subdivided plots. Each tooth was considered an experimental unit, and the average values of the sticks per tooth were considered in the analysis.^3^ The prematurely failed sticks were assigned a bond strength of zero and included in this analysis.^3^ The associations of treatment with premature failure and fracture mode were analyzed using Fisher’s exact test. The significance level for all analyses was set at 5%.

## Results

There was no significant association between premature failure and pretreatment using either etch-and-rinse or self-etch strategies at either time point (p > 0.05) (Table 2). There was a higher percentage of premature failure after 6 months of storage.

**Table 2:** Frequency (%) of sticks with premature failure according to the different strategies, dentin pretreatments, and time points

Strategy	Pretreatment	Time point	
		24 h	6 months
Etch-and-rinse	CON	0/40 (0.0%)	1/40 (2.5%)
	DMSO	1/40 (2.5%)	1/40 (2.5%)
	DMSO/ water	0/40 (0.0%)	3/40 (7.5%)
	DMSO/ ethanol	0/40 (0.0%)	2/40 (5.0%)
p-value		1.0000	0.8372
Self-etch	CON	4/25 (16.0%)	4/18 (22.2%)
	DMSO	4/23 (17.4%)	5/18 (27.8%)
	DMSO/ water	5/23 (21.7%)	4/23 (17.4%)
	DMSO/ ethanol	5/27 (18.5%)	4/20 (20.0%)
p-value		0.9837	0.8731

No significant difference was observed among the dentin pretreatments regarding microtensile bond strength for the same bonding strategy (p > 0.05) (Table 3). However, the etch-and-rinse strategy yielded higher bond strengths than did the self-etch mode (p < 0.05) at both evaluation time points. Microtensile bond strengths were lower at 6 months than at 24 h for the etch-and-rinse strategy and all dentin pretreatments (p < 0.05).

**Table 3:** Mean (standard deviation) microtensile bond strength (in MPa) according to the bonding strategy, dentin pretreatment and time point

Strategy	Pretreatment	Time point	
		24 h	6 months
Etch-and-rinse	CON	44.22 (10.20)^Aa^	34.33 (11.83)^Ba^
	DMSO	47.20 (11.75)^Aa^	31.52 (9.24)^Ba^
	DMSO/ water	44.68 (10.16)^Aa^	31.19 (10.16)^Ba^
	DMSO/ ethanol	45.52 (6.88)^Aa^	33.19 (6.38)^Ba^
Self-etch	CON	#16.64 (10.99)^Aa^	#19.96 (11.11)^Aa^
	DMSO	#22.26 (19.40)^Aa^	#18.43 (9.26)^Aa^
	DMSO/ water	#14.66 (9.10)^Aa^	#22.04 (9.18)^Aa^
	DMSO/ ethanol	#16.42 (9.37)^Aa^	#23.48 (10.87)^Aa^

Different superscript letters (capital letters in the rows and lowercase letters in the columns) indicate significant differences (p ≤ 0.05). # Differs from etch-and-rinse in the same group and time point (p ≤ 0.05). p (strategy) < 0.0001; p (pretreatment) = 0.8732; p (time) = 0.1079; p (strategy x time) = 0.1189; p (strategy x pretreatment) = 0.9250; p (group x time) = 0.2296; p (strategy x pretreatment x time) = 0.2469.

No significant association was found between the fracture mode and the dentin pretreatment (p = 0.8172) in the etch-and-rinse strategy at 24 h; the majority of sticks presented adhesive failure (Table 4). In contrast, the association was significant (p = 0.0222) at 6 months. Although most of the failures were adhesive for all experimental pretreatments (DMSO, DMSO/water or DMSO/ethanol), cohesive resin failures were predominant in the CON group.

**Table 4:** Fracture mode according to strategy, dentin pretreatment and time point

Strategy	Time point	Pretreatment	Adhesive	Cohesive in resin	Cohesive in dentin	Mixed
			n (%*)			
Etch-and-rinse	24 h	CON	22 (55.0%)	5 (12.5%)	12 (30.0%)	1 (2.5%)
		DMSO	20 (51.3%)	5 (12.8%)	12 (30.8%)	2 (5.1%)
		DMSO/ water	20 (50.0%)	5 (12.5%)	15 (37.5%)	0 (0.0%)
		DMSO/ ethanol	19 (47.5%)	9 (22.5%)	12 (30.0%)	0 (0.0%)
	p-value		p = 0.8172			
Etch-and-rinse	6 months	CON	11 (28.21%)	13 (33.3%)	9 (23.1%)	6 (15.4%)
		DMSO	19 (48.7%)	13 (33.3%)	5 (12.8%)	2 (5.1%)
		DMSO/ water	25 (67.6%)	6 (16.2%)	6 (16.2%)	0 (0.0%)
		DMSO/ ethanol	21 (55.3%)	11 (29.0%)	5 (13.2%)	1 (2.6%)
	p-value		p = 0.0222			
Self-etch	24 h	CON	13 (61.9%)	3 (14.3%)	3 (14.3%)	2 (9.5%)
		DMSO/ water	1 (5.3%)	8 (42.1%)	5 (26.3%)	5 (26.3%)
		DMSO/ ethanol	7 (38.9%)	9 (50.0%)	1 (5.6%)	1 (5.6%)
		DMSO/ water	5 (22.7%)	7 (31.8%)	0 (0.0%)	10 (45.5%)
	p-value		p = 0.0001			
Self-etch	6 months	CON	9 (64.3%)	1 (7.1%)	2 (14.3%)	2 (14.3%)
		DMSO	5 (38.5%)	2 (15.4%)	3 (23.1%)	3 (23.1%)
		DMSO/ water	9 (47.4%)	3 (15.8%)	1 (5.3%)	6 (31.6%)
		DMSO/ ethanol	8 (50.0%)	3 (18.8%)	1 (6.3%)	4 (25.0%)
	p-value		p = 0.8439			

*Percentage in rows.

As for the self-etch strategy, an association existed between the fracture mode and the pretreatment only at 24 h (p < 0.0001) (Table 4). The majority of failures for the CON group were adhesive (61.9%), whereas pretreatments with DMSO and DMSO/water had a majority of cohesive in resin failures, and pretreatment DMSO/ethanol exhibited mainly mixed failures. At 6 months, there was no significant association between failure type and dentin pretreatment (p = 0.8439).

**Fig 1:**
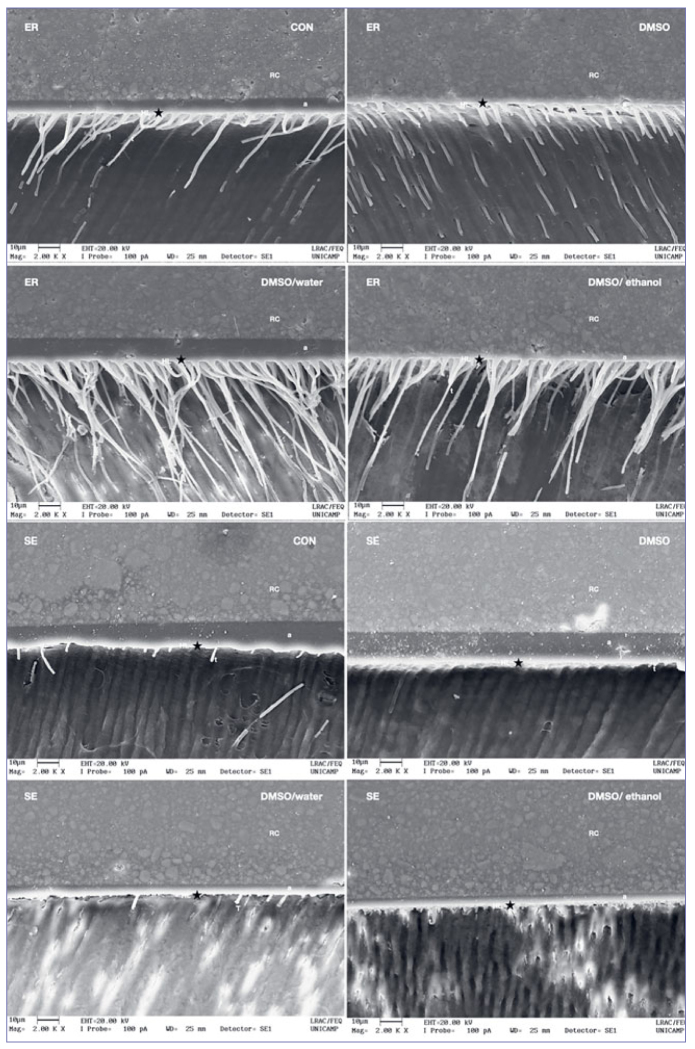
Micromorphology of the resin/dentin interface according to the bonding strategy and dentin pretreatments (2000x magnification). ER: etch-and-rinse; SE: self-etch; RC: resin composite; a: adhesive layer; t: tags; HL: hybrid layer (also indicated with a black star).

The resin/dentin interface images show that the hybrid layer had longer and more numerous resin tags when the etch-and-rinse strategy was used, and fewer, shorter tags when the self-etch mode was used (Fig 1). Micromorphological differences existed between the dentin pretreatments in each bonding strategy.

## Discussion

Simplified protocols for universal adhesives and bonding agents have been developed to achieve greater stability of the hybrid layer, by promoting more effective infiltration of the adhesive resin monomers between the collagen fibrils demineralized by the adhesive.^1,14,32^ Solutions containing DMSO have been found to be effective in enhancing the penetration ability of adhesive monomer into the dentin matrix of conventional adhesives,^21,36,39^ and increasing bond strength to dentin.^35,37,39^ However, the present study showed that DMSO (whether or not solubilized in water or ethanol) did not promote higher bond strength to dentin by using a universal adhesive with etch-and-rinse and self-etch strategies. Thus, the results failed to reject the first null hypothesis of the study.

The solvents (ethanol and water) in universal adhesives both provide and promote penetration of the adhesive’s resin monomers by eliminating dentin moisture trapped among the collagen fibers after the volatilization process.^18^ In this respect, the presence of water in the dentin layer is required to form a stable hybrid layer. On the other hand, water is also responsible for the long-term hydrolysis and deterioration of the bonded interface.^18,24^ Recognizing that the use of the etch-and-rinse strategy leaves more residual water in the hybrid layer than does the self-etch strategy,^18^ the use of pretreatments with DMSO, whether solubilized in water or ethanol or not solubilized, could lead to the replacement or displacement of residual water more effectively, in a relatively short application period.^7,34,35^ DMSO competes with water molecules in collagen interpeptide hydrogen bonding,^43^ and increases collagen interfibrillar spacing.^47^ However, the dentin pretreatment with DMSO (solubilized in water or ethanol or not solubilized) in the present study may made the evaporation of these solvents more difficult, since DMSO is a polar aprotic compound that can also absorb water, because it is characteristically attracted to hydrogen molecules.^36^ Since the amount of water and ethanol in Scotchbond Universal adhesive is about 30%, the use of a dentin pretreatment containing yet another a solvent in addition to DMSO, water and ethanol, may have hindered evaporation of the solvents in both strategies in the universal adhesive, since the hydrophobic and hydrophilic content were applied in the same procedure (unlike a conventional 3-step adhesive).^35^ Whether solubilized in water or ethanol, pretreatment with DMSO may have contributed to forming residual moisture, which not only impairs volatilization of the DMSO solvent, but fails to improve the bond strength of the respective groups using either strategy. The same outcome could be expected even if pretreatment were applied in a manner to allow volatilization of the solvents, eg, by performing circular scrubbing movements for 60 s.^35–37^

Higher microtensile bond strength was achieved at both time periods when the etch-and-rinse strategy was used, as corroborated in the studies by Wagner et al,^46^ Luque-Martinez et al,^18^ and Dačić et al.^10^ The micromorphology of the interface shows the presence of longer tags and a thicker hybrid layer, since the acid conditioning increases the surface energy and removes the smear layer.^5^ On the other hand, the universal adhesive used is classified as “ultra-mild,” with a pH of 2.92. It promotes the demineralization of superficial dentin, which allows maintaining the part of the hydroxyapatite that is bound to collagen fibers, and also chemical bonding to the functional monomer.^23,41^ Although resin tags are known not to contribute significantly to resin-dentin bonding strength,^17^ a higher frequency of premature failures can be expected when the self-etch strategy vs the etch-and-rinse strategy is used, because of the thinner hybrid layer,^10^ especially at the 24-h time point. After 6 months of storage, a higher frequency of premature failures was observed in both strategies, owing to the hydrolytic and enzymatic degradation promoted by the storage medium. This can be expected especially because of the hydrophilic character of the monomeric units of the polymers in the adhesive,^2^ including hydroxyethyl methacrylate (HEMA), which favors water sorption.^33^ After 6 months of storage, a greater number of cohesive failures in resin were also observed, as a result of hydrolytic degradation caused by water sorption.^4^ After 6 months of storage, the resin composite absorbed water and released unpolymerized monomers that did not react after photocuring. This may have compromised not only the physical and mechanical properties of the resin composite, owing mostly to the breaking of the hydrolytic bond between the silane and the inorganic particles, but also the cohesive strength of the resin.^33^

After 6 months of storage, the hydrolytic and proteolytic degradation^9^ caused by the storage medium when using the etch-and-rinse strategy was significant, resulting in lower bond strengths than those observed at 24 h. DMSO is known to decrease collagen degradation in the hybrid layer by inhibiting the activity of MMP-2 and MMP-9.^13,39^ Nevertheless, the storage medium used (SBF), containing calcium and zinc, may have promoted higher MMP activity, and consequent loss of stiffness and solubilization of the dentinal matrix collagen.^9^ This is especially relevant to the etch-and-rinse strategy, which denudes the collagen fibrils, and may provide greater enzymatic activity.^9^ When performing acid etching, smear layer removal exposes the dentin collagen, and leads to increased dentin permeability.^25^ This makes the bonding interface vulnerable to hydrolytic and enzymatic degradation.^22^ The residual water and non-evaporated solvent in the adhesive could remain trapped after polymerization, thus compromising the bond strength and the mechanical properties of the hybrid layer.^30^ However, pretreatments with DMSO did not benefit bond strength longevity (it remained the same as the control group). These results failed to reject the second null hypothesis of the present study. Regarding the self-etch strategy, the fact that bond strengths were maintained over time can be attributed to the 10-MDP functional group interacting ionically with the calcium in the dentin, and forming a relatively long 10-MDP-calcium carbonyl chain hydrophobically on the dentin surface.^40,44^ This chain plays an important role in increasing the durability of the universal adhesive dentin bond in the self-etch strategy.^15,29^ Stape et al^35^ also performed pretreatment with 50% DMSO incorporated with ethanol or water in a universal adhesive using a self-etch strategy, with no significant influence on the microtensile bond strength. This can be explained by the reduced availability of crosslinked dimethacrylate monomers in universal adhesives, which penetrate better when used with DMSO, but which have limited bond strength to the same extent as they do with a three-step etch-and-rinse adhesive. However, treatment with DMSO, whether solubilized in water or ethanol or not solubilized, influenced the failure mode when the self-etch strategy was used. This was reflected in the higher prevalence of cohesive resin failures in the self-etch group than in the control group after 24 h, thereby leading to the rejection of the third null hypothesis of the study. The DMSO groups (whether or not solubilized in water or ethanol) had a higher prevalence of cohesive resin failures, which may suggest that pretreatments with DMSO led to an improvement in hybrid layer integrity, as reported by Stape et al.^35^

Although pretreatment with DMSO, whether solubilized in water or ethanol or not solubilized, has been found to improve the encapsulation of collagen fibers as well as the quality of the collagen-resin interface,^7,13,28,34,35–37,39^ our study suggests that no benefit is derived from using DMSO as a pretreatment for a universal adhesive with either etch-and-rinse or self-etch strategies, since our study showed that there was no increase in bond strength to dentin, not even after 6 months.

## Conclusion

The use of dentin pretreatments containing DMSO did not improve the bonding or the micromorphology of a universal adhesive system in etch-and-rinse or self-etch modes.

## Clinical relevance:

DMSO (whether or not solubilized in water or ethanol) as a pretreatment for a universal adhesive system with either etch-and-rinse or self-etch strategies does not improve bond strength to dentin, not even after 6 months.
